# Dynamics of β-cardiac myosin between the super-relaxed and disordered-relaxed states

**DOI:** 10.1016/j.jbc.2025.108412

**Published:** 2025-03-19

**Authors:** Robert C. Cail, Faviolla A. Báez-Cruz, Donald A. Winkelmann, Yale E. Goldman, E. Michael Ostap

**Affiliations:** 1 Department of Physiology, Perelman School of Medicine, University of Pennsylvania, Philadelphia, Pennsylvania, USA; 2 Pennsylvania Muscle Institute, Perelman School of Medicine, University of Pennsylvania, Philadelphia, Pennsylvania, USA; 3 Department of Pathology and Laboratory Medicine, Robert Wood Johnson Medical School, Rutgers University, New Brunswick, New Jersey, USA; 4 Department of Pharmacology and Department of Molecular and Cell Biology, University of California, Davis, California, USA

**Keywords:** cardiac, disordered-relaxed, kinetics, myosin, super-relaxed

## Abstract

The super-relaxed (SRX) state of myosin ATPase activity is critical for striated muscle function, and its dysregulation is linked to cardiomyopathies. It is unclear whether the SRX state exchanges readily with the disordered-relaxed (DRX) state and whether the SRX state directly corresponds to the folded back interacting-heads motif. Using recombinant **β**-cardiac heavy meromyosin and subfragment 1, which cannot form the interacting-heads motif, we show that the SRX and DRX populations transition at a rate substantially faster than the ATP turnover rate, dependent on myosin head–tail interactions. Some mutations which cause hypertrophic or dilated cardiomyopathies alter the SRX-DRX equilibrium, but not all mutations. The cardiac myosin inhibitor mavacamten slows nucleotide release by an equal factor for both heavy meromyosin and subfragment 1, thus only indirectly influencing the occupancy time of the SRX state. These findings suggest that purified myosins undergo rapid switching between SRX and DRX states, refining our understanding of cardiomyopathy mechanisms.

Class II myosin paralogs across animalia species form a conserved “off” state in the presence of ATP, in which the two ATPase motor domains of each molecule fold back onto the coiled-coil tail domains, thereby preventing binding to the actin filament and phosphate (P_i_) and ADP release (1). The off-state conformation in striated muscle is termed the interacting-heads motif (IHM) because of interhead and head–tail interactions responsible for its stabilization (2). Nonmuscle and smooth-muscle myosin-II paralogs form a similar state, termed the 10S conformation, which is disengaged upon activation of the muscle through phosphorylation of the regulatory light chains (RLCs) (3, 4). In contrast, skeletal and cardiac muscle myosins do not require light chain phosphorylation for activation. Rather, RLC phosphorylation modulates the partition of molecules into the IHM (5, 6). The two myosin motor head domains in the IHM conformation adopt distinct conformations, with a blocked head that interacts with the myosin backbone and a free head, which binds to the blocked head (7). The conversion between open conformation and IHM/10S conformation is essential to regulating tension maintenance in nonmuscle cells, smooth muscle activation, and striated muscle force generation (8, 9, 10). Dysregulation of partitioning into the IHM is implicated in many disorders, including hypertrophic cardiomyopathy (HCM) and dilated cardiomyopathy (DCM) (11, 12).

In striated muscle, a biochemically defined population of myosin heads, termed the super-relaxed (SRX) state, saves metabolic energy in the relaxed condition (13, 14). The SRX state was identified in skinned skeletal and cardiac muscle cells using turnover of the fluorescent nucleotide N-Methylanthraniloyl-ATP (mantATP), which is hydrolyzed to mantADP and then released from the myosin heads in two distinct kinetic populations, adequately fitted by exponential decays: a faster releasing component (at ∼0.05 s^−1^), representing a population termed the disordered-relaxed (DRX) state, and the ∼10-fold slower SRX component (14, 15). X-ray diffraction studies have correlated the SRX and DRX states to myosin heads that are proximal or more distal, respectively, to the backbone of the thick filament (9, 16). The myosin heads that participate in any given contraction are postulated to come from the DRX population. Cardiac SRX heads are thought to stay detached until a regulatory event, such as phosphorylation or length-dependent activation, causes them to shift into the DRX group (9, 14, 15, 16, 17).

Are the SRX and IHM states the same? Investigators performing single-turnover kinetic experiments employing purified myosin, either as the dimer-forming heavy meromyosin (HMM, which is capable of forming the IHM state) or even the head-only subfragment 1 (S1, which is incapable of entering the IHM state) have reported similar results to skinned striated muscle. Single-nucleotide turnover rates for purified myosins are similar to those of skinned myocytes, with double-exponential fits to mantATP turnover taken to measure the relative proportion and kinetics of SRX and DRX nucleotide turnover (18, 19, 20, 21). Single-nucleotide turnover is slower for HMM than for S1, indicating that the presence of the S2 tail impacts ATPase activity (18). These results suggest that IHM myosin molecules are in the SRX state. Direct measurement of the kinetics of an SRX-DRX transition, though, has proved difficult (20). Additionally, X-ray diffraction experiments on myofibrils and FRET studies of purified myosins have undermined the equivalence of SRX with IHM in some conditions, finding evidence for slow-phase nucleotide release that is not correlated to proximal IHM heads (11, 20, 21, 22). Moreover, the consistent presence of a measurable slow phase of turnover in S1 is confounding for the head–tail interactions thought to be central to formation of IHM (19, 20, 21). A recent publication has reported that a single-exponential decay function is sufficient to model nucleotide release, challenging the reliability of earlier mantATP experiments that were interpreted as the double exponential decay representing the IHM and the open (non-IHM) conformations (23).

The two β-cardiac myosin heads in the IHM conformation are unambiguously in the prepowerstroke (PPS) state with ADP.P_i_ in each of the active sites (7). This configuration presumably cannot release P_i_, as the P_i_ release tunnel is blocked by the lever arm. Thus, to undergo P_i_ and nucleotide release, purified myosin molecules seemingly must exit the IHM, with a reaction according to Reaction 1.

$$\text{SRX}-{M.\text{ADP}}^{**}.P_{i}\;\begin{array}{c} \overset{k_{+1}}{\to }\\ \underset{k_{-1}}{\leftarrow } \end{array}\text{DRX}-{M.\text{ADP}}^{**}.P_{i}\;\overset{k_{\text{DRX}}}{\to }M+P_{i}+.{\text{ADP}}^{*}$$

### Reaction 1

Where M is myosin, ADP∗∗ is the high-fluorescence mantADP in the active site, and ADP∗ is the low-fluorescence mantADP in solution.

Whether the IHM (and by implication, the SRX state) is in a rapid equilibrium with the open/DRX state, or whether the two populations are distinct because of a slow transition between the SRX and DRX states, is not yet known (Fig. 1*A*). A double-exponential curve for mantATP turnover suggests that the SRX and DRX populations are separated by a kinetic transition out of the SRX state that is slower than the basal ATPase rate, with this SRX-DRX transition as the rate-limiting step for slow nucleotide release. In the case that the equilibrium between the two states is rapid relative to product release, the overall rate of nucleotide release, *k*_obs_, is expected to follow single-exponential kinetics, with an observed rate given by

(1) *k*_obs_ = *k*_DRX_ · (*k*_+1_/*k*_-1_) = *k*_DRX_ · *K*_EQ_,

where *k*_DRX_ is the elementary product release rate, *k*_+1_ and *k*_-1_ = interconversion rates between SRX and DRX (Reaction 1), and *K*_EQ_ = the equilibrium constant for the SRX to DRX isomerization.

**Figure 1:**
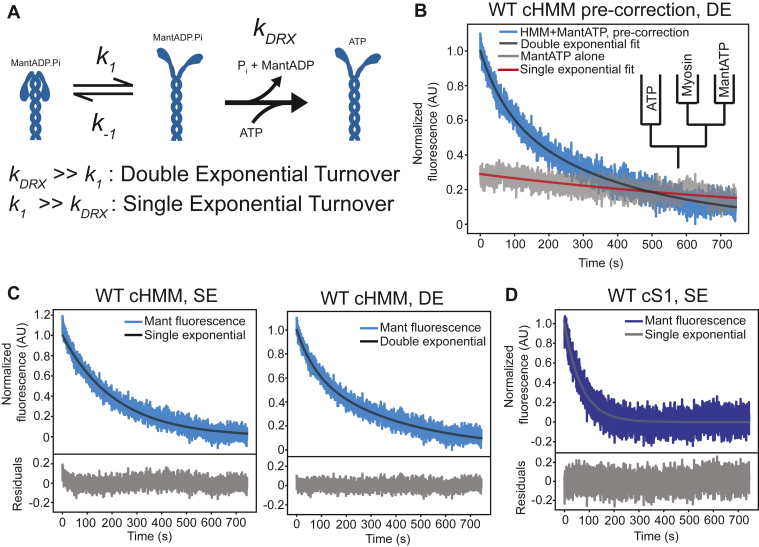
**Single- *versus* double-exponential equations fitted to mantATP nucleotide turnover data.***A*, reaction scheme of SRX-DRX transition followed by release of nucleotide as MantADP. A fast *k*_1_ relative to *k*_DRX_ results in single phase kinetics for nucleotide release rate limited by *k*_DRX_; if *k*_1_ is substantially slower than *k*_DRX_, the fluorescence decay would be biphasic, with the slow phase rate limited by *k*_1_. *B*, single-nucleotide turnover of mantATP from the active site of WT-cHMM (*blue*) with double-exponential fit, along with a trace of mantATP in the absence of myosin (*gray*) demonstrating a significant amplitude and rate of mant fluorescence decrease (photobleaching) during kinetic acquisition. Inset: schematic of double-mixing stopped-flow apparatus for experimental conditions employed throughout this paper. *C*, WT-cHMM single-nucleotide turnover after correction for mant photobleaching. *Left:* double-exponential fit with residuals. *Right:* single-exponential fit with residuals. *D*, WT-cardiac subfragment-1 (-cS1) single-nucleotide turnover, corrected for mant photobleaching, with single-exponential fit and residuals. cHMM, cardiac heavy meromyosin; DE, double exponential; mantATP, N-Methylanthraniloyl-ATP; SE, single exponential.

HCM-causing mutations in *MYH7* (encoding the principal ventricular myosin paralog β-cardiac myosin), *MYLK3* (cardiac-specific RLC), and *MYBPC3* (cardiac myosin-binding protein C) have been proposed to decrease the number of myosins in SRX, thereby exhibiting an increase in the fast (DRX) amplitude in double-exponential fits and a hypercontractile state that leads to hypertrophy (24, 25, 26). However, it is not clear if disruption of the SRX-DRX distribution is the major driver for disease, as mutations in *MYH7* can also influence other mechanical and biochemical parameters of myosin’s function, such as working stroke and actin affinity (24, 25, 26, 27, 28).

In the current work, we employed purified WT and HCM mutant cardiac heavy meromyosin (cHMM) and S1 constructs to investigate 1) the kinetic switching and/or equilibrium state represented by single-nucleotide turnover product release and 2) how head–tail interactions, myopathy mutations, and small-molecule treatments might alter the SRX/DRX partition.

## Results

### MantATP turnover follows single-exponential kinetics for HMM and S1

We measured the single-nucleotide turnover rate of purified, two-headed, cHMM using stopped-flow fluorimetry. In this assay, nucleotide-free myosin is mixed with 1.1-fold excess of mantATP, aged for 10 s to allow nucleotide binding and hydrolysis, and then chased with 1 mM unlabeled ATP to outcompete mant nucleotide as it is released from the myosin active site which decreases the fluorescence intensity (Fig. 1*B*, inset). Under the experimental conditions in the absence of actin, the rate-limiting transition is P_i_ dissociation from the myosin·ADP·P_i_ complex. ADP is released promptly thereafter which generates the observed signal. We employed 295-nm excitation, which excites the mant fluorophore mainly via FRET from Tryptophan 507 near β-cardiac myosin’s nucleotide-binding pocket (26).

Single-turnover experiments performed with WT β-cHMM resulted in a biphasic decrease in fluorescence intensity that was well fitted by a double-exponential decaying function, with a slow phase (*k*_s_ = 0.0032 ± 0.0002 s^−1^) contributing a larger amplitude (*A*_s_ = 0.80 ± 0.05) than the fast phase (*k*_f_ = 0.016 ± 0.003 s^−1^; *A*_f_ = 0.20 ± 0.05) (Figs. 1*B*, S1*B*, Table S1). As the excitation spectrum of mantATP extends to 295 nm (Fig. S1*A*), we performed control experiments to determine if changes in fluorescence emission occur independently of myosin kinetics, for instance due to photobleaching. Indeed, over the acquisition window, mantATP fluorescence in the absence of myosin was decreased which we attribute to photobleaching (Figs. 1*B*, S1*B*). The rate of photobleaching, at 0.0017 ± 0.0002 s^−1^, is similar to the slow-phase rate reported above. Because the direct excitation of mantATP at 295 nm is less than the FRET excitation occurring in the presence of myosin, this is expected to be a modest underestimate of the mant photobleaching during myosin single turnover. Single-turnover transients were corrected for photobleaching by subtracting the fluorescence values of a control trace of mantATP alone, collected each day under the same experimental conditions (buffers and instrument settings) as myosin experimental traces. Photobleaching-corrected transients were well fitted by a single-exponential function with rate of *k*_obs_ = 0.0047 ± 0.0005 s^−1^ (Fig. 1*C*, Table 1). A two-exponential fit to the corrected transient was not statistically justified according to the Bayesian information criterion and the log-likelihood ratio test (Fig. S2, Table S2) (29, 30).

**Table 1:** Single-turnover kinetics and equilibrium constant for WT and myopathy mutant HMMs

Myosin type	Corrected mant-ADP release rate	Papain-digested mant-ADP release rate	*K*_eq_
WT-cHMM	0.0047 ± 0.0005 s^−1^	0.013 ± 0.001 s^−1^	0.32 ± 0.03^a^ 0.33 ± 0.05^b^
WT-cS1	0.0148 ± 0.0002 s^−1^	N/A	N/A
E497D-cHMM	0.029 ± 0.003 s^−1^	0.067 ± 0.003 s^−1^	0.49 ± 0.04^b^
R712L-cHMM	0.015 ± 0.004 s^−1^	0.05 ± 0.02 s^−1^	0.3 ± 0.1^b^
S532P-cHMM	0.0035 ± 0.0003 s^−1^	0.011 ± 0.002 s^−1^	0.32 ± 0.05^b^

cHMM, cardiac heavy meromyosin; HMM, heavy meromyosin; mant, N-Methylanthraniloyl.

Data are reported as mean ± SD.

a Calculated assuming *k*_*DRX*_ is the rate obtained from the WT-cS1 single-turnover experiment.

b Calculated using *k*_*DRX*_ is the rate obtained from the papain-digested cHMM.

mantATP single-turnover assays performed with single-headed WT cardiac motor domain (WT-cS1), which is incapable of forming dimers or motor–S2 interactions, resulted in fluorescence transients that were best fit to a single-exponential function with a rate of *k*_obs_ = 0.0148 ± 0.0002 s^−1^ after correcting for photobleaching (Fig. 1*D*, Table 1). This rate is > 3-fold faster than observed for WT-cHMM (Table 1), and it likely represents the rate of ATP turnover equivalent to the DRX state of myosin (*k*_DRX_).

The faster product release from WT-cS1, coupled with the single exponential decay of fluorescence with WT-cHMM contradicts the model assuming slow escape from SRX (Fig. 1*A*, “slow transition” model). Such a slow transition out of the SRX state (*k*_1_ << 0.015 s^−1^) would result in a two-exponential mantATP transient. Rather, the results are compatible with the “fast transition” (*k*_1_ >> 0.015 s^−1^) model in Figure 1*A* including exchange between SRX and DRX that is rapid relative to the product release step, thus generating a single exponential decay of fluorescence given by Equation 1 above (Fig. 1*A*; Table S2) (23).

If we accept that the SRX and DRX states are in equilibrium, we model the SRX-DRX as a rapid equilibrium with the ATP hydrolysis and product release rates of the DRX state equivalent to that determined for WT-cS1. We calculate the equilibrium constant for the SRX to DRX transition using the observed turnover rate of WT-cHMM (*k*_obs_) as in Equation 1. *k*_DRX_ is taken as the ADP release rate from the DRX state (equivalent to S1 release rate), and *K*_EQ_ is the equilibrium constant of the SRX to DRX transition. This calculation results in an apparent equilibrium constant, *K*_EQ_ = 0.32 ± 0.03 (Table 1). This procedure is conceptually similar to the long-tail/short-tail ATPase ratio assay employed by other groups, in which actin-activated ATPase activity is measured in the presence or absence of the IHM-forming proximal S2 domain, demonstrating that head–tail interactions reduce the steady-state ATPase activity by approximately 50% (19, 21, 26, 31).

### Intact HMM is required for slowed nucleotide release

**Figure 2:**
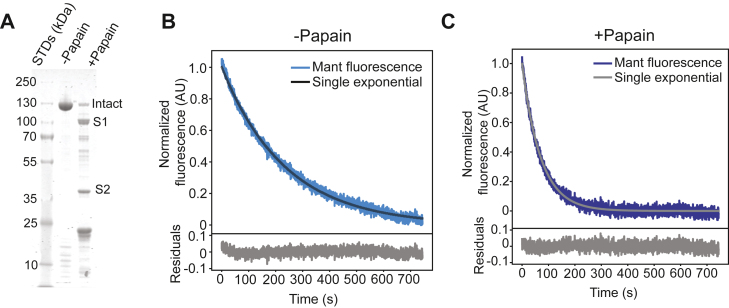
**Digestion of WT-cHMM to produce S1 and S2 speeds nucleotide release.***A*, coomassie stained SDS-PAGE gel of WT-cHMM demonstrates digestion by papain protease into S1, S2, and other fragments. *B*, undigested control samples incubated without the protease have single-turnover kinetics that are well fitted by a single-exponential function, similar to untreated WT-cHMM. *C*, upon papain digestion for 5 min, the single-turnover rate increases significantly to that of WT-cS1, as expected for myosin heads incapable of forming head–tail interactions. cHMM, cardiac heavy meromyosin.

We further tested whether IHM interactions in the WT-cHMM molecule are required to slow the single-turnover experiments by performing limited proteolytic digestion of the WT-cHMM with papain, which cleaves myosin at the heavy chain hinge just past the RLC binding IQ motif, releasing S1 (32). Papain digestion was quenched by addition of the irreversible protease inhibitor E-64 (see Experimental Procedures). SDS-PAGE revealed the proteolytic fragments formed by papain treatment, with predominant bands running at the positions expected for S1, S2, and intact single-headed S1-S2 molecules, none of which are capable of forming IHM (Fig. 2*A*). In this assay, the measured rate of mantATP turnover is independent of the starting concentration of active myosin heads as long as the reaction is chased with saturating unlabeled ATP. Thus, differences in recombinant protein quality and the presence of inactive myosin heads should not impact the measured rates, unlike measurements of steady-state ATPase normalized per head (31).

The mantATP turnover rate of papain treated WT-cHMM (0.013 ± 0.001 s^−1^) was similar to WT-cS1, with papain-free control samples (incubated in digestion buffer having identical composition but without papain) retaining the slower single-turnover kinetics (*k*_obs_ = 0.0042 ± 0.0004 s^−1^) observed in untreated samples (Fig. 2, *B* and *C*). The equilibrium constant for digested *versus* undigested samples, according to Equation 1, is 0.33 ± 0.05, similar to that of WT-cHMM *versus* WT-cS1 (Table 1). A double-exponential mantATP transient was observed at shorter papain incubation times in some experiments, likely indicating the presence of uncleaved WT-cHMM molecules capable of forming the IHM (Fig. S3). Since the cleaved and uncleaved populations would not be in equilibrium, the observed transient with two distinct kinetic components is expected. The results indicate that the IHM-forming interactions between myosin heads and proximal tail domains are essential for the rapid equilibrium that slows nucleotide release in WT-cHMM. While our data suggest that the SRX and IHM states are directly correlated under our experimental conditions with purified proteins, data from other studies suggest possible uncoupling of these states (11, 17, 18, 19, 20, 21, 22).

### Cardiomyopathy mutations can directly alter the SRX/DRX equilibrium, but this is not universal

Certain MYH7 mutations linked to familial HCM or DCM are thought to alter the proportion of myosin heads in the IHM/SRX state, with some mutations decreasing it in HCM and increasing it in DCM. This shift may impact force production and cooperative thin filament activation; however, it remains unclear whether all MYH7 mutations follow this pattern (24, 25, 26, 27). Published experiments with purified mutant cHMM revealed changes in mantATP transients that have been interpreted to indicate alteration of the SRX-DRX equilibrium for many mutations (25, 26). Thus, we investigated the impact of select cardiomyopathy mutations on SRX-DRX equilibrium with the appropriate corrections for photobleaching.

We examined the single-turnover kinetics with cHMM constructs containing HCM mutations (E497D, R712L) and a DCM mutation (S532P). All three mutations demonstrated single-exponential turnover (Fig. S4). E497D-cHMM and R712L-cHMM both increased the rate of nucleotide release relative to WT-cHMM, with rates of 0.029 ± 0.003 s^−1^ and 0.015 ± 0.004 s^−1^, respectively (Table 1). S532P-cHMM demonstrated little change relative to WT-cHMM (*k*_obs_ = 0.0035 ± 0.0003 s^−1^, Table 1). Thus, some but not all of these cardiomyopathy mutations change the basal rate of nucleotide release with S1–S2 interactions intact.

**Figure 3:**
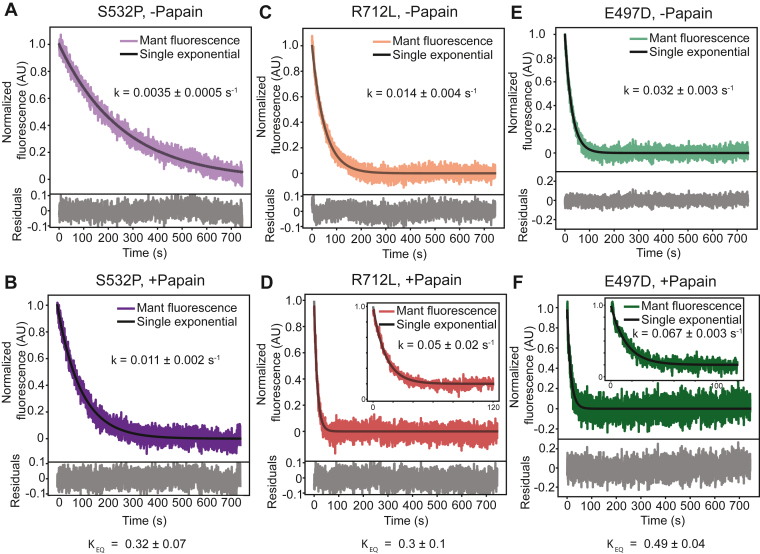
**Mutant-cHMM molecules increase single-turnover rate upon proteolytic digestion with papain.***A* and *B*, turnover rate for S532P-cHMM increases from 0.0035 ± 0.0005 s^−1^ to 0.011 ± 0.002 s^−1^ upon papain digestion, resulting in a *K*_EQ_ of 0.32 ± 0.07, similar to WT. *C* and *D*, turnover rate for R712L-cHMM increases from 0.016 ± 0.004 s^−1^ to 0.05 ± 0.02 s^−1^ upon papain digestion, resulting in a *K*_EQ_ of 0.3 ± 0.1, similar to WT. Inset: turnover of papain-digested R712L on a faster time-base to show single-exponential fit for early time points. *E* and *F*, turnover rate for E497D-cHMM increases from 0.032 ± 0.003 s^−1^ to 0.067 ± 0.003 s^−1^ upon papain digestion, resulting in a *K*_EQ_ of 0.49 ± 0.04, significantly higher than WT. Inset: turnover of papain-digested E497D on a faster time-base to show single-exponential fit on early time points. Data are reported as mean ± SD. cHMM, cardiac heavy meromyosin.

Upon papain digestion, all three mutated myosins had increased rates of nucleotide release. Paired undigested cHMM controls, treated in digestion buffer lacking papain, demonstrated the same product release rate as untreated samples (Fig. 3, *A*, *C* and *E*). Upon papain digestion, the rate constants for E497D-cHMM and R712L-cHMM increased to 0.067 ± 0.003 s^−1^ and 0.05 ± 0.02 s^−1^, respectively, while S532P-cHMM increased to 0.011 ± 0.002 s^−1^ (Fig. 3, *B*, *D* and *F*, Table 1). With these values, we calculate equilibrium constants for SRX-DRX equilibrium at 0.49 ± 0.04, 0.3 ± 0.1, and 0.32 ± 0.05 for E497D, R712L, and S532P, respectively (Table 1). Thus, E497D alters SRX-DRX equilibrium, but R712L and S532P do not alter it.

### *Mavacamten slows nucleotide release without affecting K*_EQ_*for SRX-DRX*

The drug mavacamten (mava), recently approved to treat obstructive HCM, is well established as an inhibitor of myosin activity in intact muscle cells, skinned muscle fibers, and isolated myosin thick filaments (33, 34, 35). It has been proposed to function in part by stabilizing SRX by sequestering heads in the “off” state (21, 35, 36). Mava is also a potent inhibitor of S1 activity in the absence of the S2; thus, it may work at multiple levels to inhibit myosin function and decrease thin filament activation (21).

**Figure 4:**
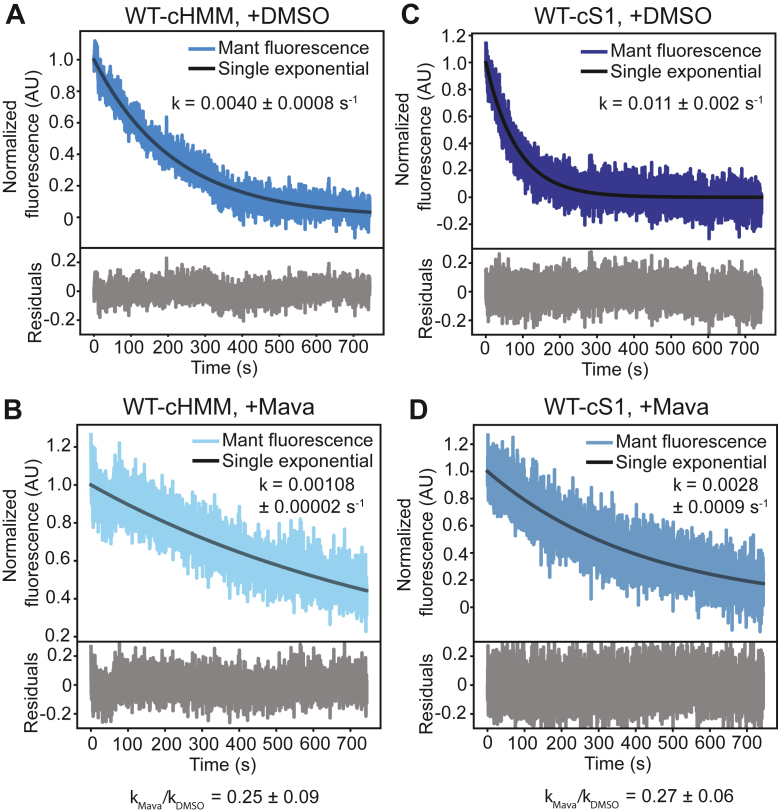
**Treatment with mavacamten slows nucleotide turnover by an equal percentage for WT-cHMM and WT-cS1.***A* and *B*, WT-cHMM treated with DMSO (*A*) or 10 μM mava (*B*) showing a 4-fold slowing of nucleotide release in the presence of mava. *C* and *D*, WT-cS1 treated with DMSO (*C*) or 10 μM mava (*D*), also showing a 4-fold slowing of nucleotide release in the presence of mava. The *K*_EQ_ for the SRX/DRX equilibrium in the presence of mavacamten is 0.38 ± 0.07, statistically indistinguishable from untreated samples. Data are reported as mean ± SD. cHMM, cardiac heavy meromyosin.

We performed single-nucleotide turnover mantATP experiments with WT-cHMM and WT-S1 constructs to test mava’s effects on the *K*_EQ_ for SRX-DRX. Mava (10 μM) decreased mantATP turnover by cHMM 3.7-fold compared to a dimethyl sulfoxide (DMSO)-only vehicle control, as reported previously (Fig. 4, *A* and *B*, Table 2) (21). A nearly identical 3.9-fold inhibition of WT-S1 was observed (Fig. 4, *C* and *D*, Table 2). These results indicate that mava does not substantially change the apparent equilibrium constant for the SRX/DRX transition (*K*_EQ_ = 0.38 ± 0.07) (Table 2) under these conditions. Instead, as with some of the myopathy mutations, it appears to alter ATPase kinetics rather than SRX-DRX equilibrium.

**Table 2:** Single turnover in the presence of DMSO or mavacamten

Myosin type	Corrected mant-ADP release rate, DMSO	Corrected mant-ADP release rate, mava	*K*_eq_, DMSO	*K*_eq_, mava
WT-cHMM	0.0040 ± 0.0008 s^−1^	0.00108 ± 0.00002 s^−1^	0.36 ± 0.09	0.38 ± 0.07
WT-cS1	0.011 ± 0.002 s^−1^	0.0028 ± 0.0009 s^−1^	NA	NA

Data are reported as mean ± SD.

cHMM, cardiac heavy meromyosin; mant, N-Methylanthraniloyl.

## Discussion

We propose that single-turnover mantATP transients acquired in the presence of cHMM are best described by an essentially single-exponential process and that HMM molecules exist in a rapid equilibrium between SRX and DRX. The structure of the IHM state prevents nucleotide exchange, so the dynamic partitioning of motors into the IHM state leads to slower nucleotide turnover, as described in Equation 1. Our findings support previous observations of faster nucleotide release by S1 myosins than HMM, but they contradict a recent report of a single-exponential curve for HMM nucleotide release at the same rate as S1 (23). These discrepancies might be attributed to differences in protein preparations (21, 23).

### SRX-DRX in the heart

It is likely that the kinetics of the SRX-DRX transition of myosin *in situ* are slower than the rapid equilibrium kinetics we observed with purified cHMM. Cryo-electron tomography studies revealed that myosin in the intact cardiac thick filament forms three distinct classes templated by unique head/tail positions and interactions with MyBP-C that are not present in purified cHMM (37, 38, 39). Therefore, in cardiac muscle, there may exist a kinetically sequestered state that is not attained with cHMM. Indeed, the initial studies that identified a slow turnover population via mantATP were performed with cardiac myocytes (14, 15).

The slow-exchanging SRX myosins act as a motor reservoir ready to be activated in response to requirements for higher cardiac output. If the transition of SRX motors to the DRX state occurs at a rate slower than 0.05 s^−1^, as suggested by double exponential fits of single-turnover experiments, then recruitment of the motors would take place on a scale tens or hundreds of seconds which is not compatible with rapid changes in cardiac output. Activation of SRX heads may be the result of RLC phosphorylation, as observed for nonmuscle and smooth myosins (4, 5). However, the Frank-Starling mechanism, powered by length-dependent and stretch-activated muscle output, exhibits a beat-by-beat response, faster than light chain phosphorylation, to increase force production [reviewed in (40) and (41)]. This activation may be the result of the interaction of MyBP-C with myosin, and the presence of the rapidly exchanging SRX-DRX states identified in this study are likely important in this regard.

### Relationship of mava to the SRX-DRX equilibrium

Mava does not change the SRX-DRX equilibrium of WT-cHMM under the conditions of the present experiments (Fig. 4). However, it does slow nucleotide release from the DRX state. Structural studies reveal that mava stabilizes the PPS state by interacting with the N-terminal subdomain of the myosin head and the converter, resulting in a substantial slowing of phosphate release from the active site (34). Importantly, mava does not interact with IHM-forming elements in the region of the motor domain known as the mesa, upper 50 KDa subdomain, or proximal S2 tail. If mava were to increase SRX, we propose that it does so by increasing the proportion of myosins in the PPS that would redistribute into the SRX-DRX equilibrium, rather than changing the elementary SRX-DRX equilibrium constant directly. Thick-filament IHM structures have been solved in the presence of mava, consistent with an enhancement of PPS myosin heads induced by slowed phosphate release (37); however, there may be additional biochemical factors in the thick filament that alter mava interactions not captured with purified HMM structures or biochemical studies (34).

The equilibrium constant for the elementary ATP cleavage step by β-cardiac myosin favors the posthydrolysis state, and the rate-limiting step for nucleotide exchange in the absence of actin is phosphate release (42). This means that most of the motor domains during the ATPase cycle in the absence of actin are in the PPS state. Changes to the equilibrium constant of ATP hydrolysis will change the number of heads in the PPS state, altering the rate of single-nucleotide turnover; heads in the PPS state will rapidly form an equilibrium between SRX and DRX conformations. Given this distribution, it is not surprising that the SRX-DRX equilibrium for HMM seems unaffected by mava. These results are largely consistent with previous studies on the effect of mava, which have found at most a modest (4%) difference between IHM and open conformations in the presence of mava, with mava-stabilized heads still available for thin filament interactions upon inotropic stimuli (20, 36).

### The SRX-DRX equilibrium and disease

HCM and DCM are sometimes described as diseases of hypercontractility and hypocontractility, respectively, because of their effects on systole, diastole, and left ventricular ejection fraction (25). However, for purified myosins or isolated myofibrils, the effects on force production, kinetic transitions, and tension maintenance are conflicting: some mutations follow the hypercontractile/hypocontractile phenotype, while others do not (26, 27, 28, 29). Destabilizing or stabilizing the IHM is an attractive unifying hypothesis for generating HCM or DCM effects on contractility, irrespective of the mechanochemical changes to individual myosins (25, 29). Our results imply that this model is not universal, but rather the SRX-DRX equilibrium is one of several contributing factors. Cardiomyopathy mutations can change other kinetic parameters such as the nucleotide release rate or the equilibrium constant for ATP hydrolysis ([M.ADP.Pi]/[M.ATP]), which must be considered as possible contributions to disease etiology. For HCM-mutant myosins that reduce the equilibrium constant for ATP hydrolysis, mava would be expected to increase the proportion of posthydrolysis, PPS myosin molecules (M.ADP.Pi), thus increasing the number of SRX heads irrespective of the SRX/DRX equilibrium constant. Importantly, a parsimonious explanation of mava’s effect is sufficient to explain its utility in treating obstructive HCM; since HCM is a disease of hyper-contractility, inhibition by mava alleviates the symptoms.

## Experimental procedures

### Protein purification

A HMM (cHMM) or subfragment-1 (cS1) construct of human β-cardiac myosin (MHY7) was expressed in C2C12 myoblasts and purified as previously described (43). The cHMM or cS1 coding DNA was cloned into the pShuttle-IRES-hrGFP-1 vector (Agilent Tech) and an Ad-Myo-Flag virus was prepared and amplified for expression of cHMM/cS1 protein in C2C12 cells. The cHMM protein has 1146 residues that include residues 1 to 1138 of the human MYH7 gene and a FLAG tag on the C terminus (res. 1139–1146). cHMM point mutants were generated by Genewiz. The cS1 protein has residues 1 to 787 of the human MYH7 gene, a 4-residue linker, and residues 5 to 238 of *Aequorea victoria* GFP. A FLAG-tagged variant of chimeric protein was prepared by mutating the C-terminal–coding sequence of the GFP domain from DELYK to DYKDHD. Bound light chains are those that are constitutively expressed in the C2C12 cells (MLC1/MLC3 and rLC2).

Confluent C2C12 myoblasts were infected with replication defective recombinant adenovirus (AdcHMM-Flag) at 2.7 × 108 pfu⋅ml^−1^ in fusion medium (89% Dulbecco's modified Eagle's medium, 10% horse serum, 1% fetal bovine serum). Expression of recombinant cHMM was monitored by accumulation of coexpressed GFP fluorescence in infected cells. Myocyte differentiation and GFP accumulation were monitored for 216 to 264 h after which the cells were harvested. Cells were chilled, media removed, and the cell layer was rinsed with cold PBS. The cell layer was scraped into Triton extraction buffer: 100 mM NaCl, 0.5% Triton X-100, 10 mM imidazole pH 7.0, 1 mM DTT, 5 mM MgATP, and protease inhibitor cocktail (Sigma). The cell suspension was collected in an ice-cold Dounce homogenizer and lysed with 15 strokes of the tight pestle. The cell debris in the whole-cell lysate was pelleted by centrifugation at 17,000*g* for 15 min at 4 °C. The Triton soluble extract was fractionated by ammonium sulfate precipitation using sequential steps of 0 to 30% saturation and 30 to 60% saturation. The cHMM precipitates between 30 and 60% saturation of ammonium sulfate. The recovered pellet was dissolved in and dialyzed against 10 mM imidazole, 150 mM NaCl, pH 7.4 for affinity purification of the FLAG-tagged cHMM on M2 mAb-Sepharose beads (Sigma). Bound cHMM was eluted with 0.1 mg⋅ml^−1^ FLAG peptide (Sigma). Protein was concentrated and buffer exchanged on Amicon Ultracel-10K centrifugal filters (Millipore), dialyzed exhaustively into 10 mM Mops, 100 mM KCl, and 1 mM DTT before a final centrifugation at 300,000*g* for 10 min at 4 °C. Aliquots were drop frozen in liquid nitrogen and stored in vapor phase at −147 °C. SDS-PAGE, followed by Coomassie staining and destaining, was performed according to standard procedures using 4 to 20% precast gradient gels (Novex, Invitrogen). Gels were then imaged using a LICOR Odyssey Imager.

### Acquisition of fluorescence transients

A stopped-flow apparatus in sequential mode (SX20 Stopped Flow Spectrometer) was used to acquire all transients for single-nucleotide turnover. The dead time of the instrument is < 3 ms with a total 400-μl sample volume. Fluorescence excitation was provided by a 100-W Hg lamp, where MantATP was excited by FRET from myosin W508 residue, and nucleotide fluorescence was monitored at 295 nm using a 400 nm long-pass filter for WT and mutant HMM samples. For WT S1-GFP, the same 295 nm was monitored using a 430 to 470 nm bandwidth filter.

All the reagent concentrations reported are postmixing. For single-nucleotide turnover for WT and mutant HMM, and WT S1-GFP, 50 nM myosin heads were preincubated with 52.5 nM MantATP in the aging loop for 10 s to allow nucleotide binding and hydrolysis, followed by mixing with 1 mM unlabeled ATP. All proteins and nucleotides were dissolved in KMg25 buffer. For proteolytic fragment formation from HMM to S1, 0.5 mg/ml HMM (preincubated with 0.1 U/ml of apyrase-VII on ice for 30–60 min before use) was incubated in a total of 60 μl KMg25 with 5 mM cysteine and 18.1 μM papain (diluted from 1.09 mM stock, Sigma-Aldrich p3125) for 3 min for mutant HMM and 5 min for WT HMM at room temperature. The reaction was quenched with addition of 25 μM E-64 (Cayman Chemical, 10007963); in parallel, a sample was prepared identically but without the addition of papain, to test samples and the effect of papain digestion and subsequent E-64 quenching. From the 60 μl reaction, 15 μl was saved for SDS-PAGE gel analysis, and the remaining was used for stopped-flow.

Samples were incubated on ice until single-nucleotide turnover, which was performed as mentioned above for untreated samples. Mava (10 uM) or an equal amount of DMSO was added to all reagents approximately 5 min prior to stopped-flow experiments involving these drugs. Stopped-flow data were acquired using Pro Data-SX software (www.photophysics.com/products/stopped-flow), and fitted to exponentials by a nonlinear least-squares curve fitting. Plots were made using custom python scripts. Statistics were computed in python or Microsoft Excel. Data were collected from three biological replicates for WT-cHMM and two biological replicates for WT-cS1, S532P-cHMM, E497D-cHMM, and R712L-cHMM. Three technical replicates were performed for all experiments. All values are reported as mean ± SD.

### Calculation of *K*_EQ_ for cHMM

The equilibrium constant (*K*_EQ_) for the DRX-SRX transition for cHMM was calculated using Equation 1 as follows (1): The observed rate (*k*_obs_) of mantATP release by cHMM, indicating the first single ATPase turnover after adding MgATP was determined by stopped-flow fluorometry. Transients were corrected for photobleaching by subtracting the fluorescence values of control traces of mantATP alone, collected each day under the same experimental conditions (buffers and instrument settings), but in the absence of cHMM (2). The rate of mantATP turnover by cS1 (*k*_DRX_) was determined by stopped-flow fluorometry after correcting for photobleaching. For these experiments, cS1 proteins were purified from C2C12 cells expressing cS1 constructs or obtained from cHMM by papain digestion (3). Assuming Reaction 1 and Equation 1, the equilibrium constant for the SRX-DRX transition was calculated as *K*_EQ_ = *k*_obs_/*k*_DRX_.

## Data availability

Representative traces from each experiment and paired, unedited SDS-PAGE/Coomassie gels are presented in this article; all fits and statistics are reported. Raw data is available upon request from R. C. C. and E. M. O.

## Supporting information

This article contains supporting information.

## Conflict of interests

The authors declare that they have no conflicts of interests with the contents of this article.
